# Implementation of a Digital Health Intervention (CHAMP) for Self-Monitoring of Hypertension: Protocol for 3 Interlinked Implementation Studies

**DOI:** 10.2196/72942

**Published:** 2025-10-17

**Authors:** Laura Martinengo, Ngoc Huong Lien Ha, Eugene Tay, Shao Chuen Tong, Nick Sevdalis

**Affiliations:** 1 Centre for Behavioural and Implementation Science Interventions Yong Loo Lin School of Medicine National University of Singapore Singapore Singapore

**Keywords:** chatbots, artificial intelligence, AI, digital health, hypertension, chronic disease management, implementation science, barriers, facilitators, implementation strategies

## Abstract

**Background:**

Hypertension affects 31% of the global adult population. Artificial intelligence–based chatbots may support self-management of hypertension and other chronic disorders. Chronic Disease Management Program (CHAMP) is a digital health intervention designed to support chronic disease self-management, comprising a patient-facing chatbot and an artificial intelligence–augmented clinical decision support system linked to electronic medical records.

**Objective:**

This project aims to optimize the deployment of CHAMP across primary care centers by developing implementation strategies and pilot-testing their appropriateness and effectiveness.

**Methods:**

We report 3 interlinked studies. Study 1 is a *rapid evidence review* to evaluate the factors influencing the implementation of mobile health and chatbot interventions in health care settings and the strategies and processes involved. We will follow the Cochrane Rapid Reviews Methods Group methodology and use the Consolidated Framework for Implementation Research and the unified theory of acceptance and use of technology frameworks to analyze the data. Study 2 is *a formative, mixed methods study* to inform the state of CHAMP deployment to date, the organizational structure of primary care centers, and the barriers and facilitators influencing the implementation and scale-up of CHAMP in primary care centers. We will interview members of the CHAMP development and initial implementation team, health care providers (HCPs), primary care centers’ leadership, and patient users and nonusers of CHAMP; conduct 1-day on-site visits to primary care centers; and assess the readiness to change among HCPs using validated questionnaires. Study 3 involves the *development of implementation strategies for the implementation of CHAMP in primary care centers.* We will develop a process map informed by the findings of studies 1 and 2 to outline the patient journey; map the barriers and facilitators influencing the implementation of CHAMP; and develop a set of implementation strategies to effectively implement CHAMP using the Expert Recommendations for Implementing Change taxonomy to define the implementation strategies and the action, actor, context, target, and time framework to define the strategy implementation processes. In parallel, we will use evidence-based behavioral science to test variations of the chatbot’s messages to increase patient engagement.

**Results:**

To date, the evidence review protocol (study 1) has been registered on PROSPERO. We have completed title and abstract screening and are in the full-text screening phase. For study 2, we obtained ethics approval and conducted the semistructured interviews with HCPs, primary care centers’ leadership, and members of the CHAMP development and initial implementation team. We are awaiting institutional review board approval to start the interviews with patients.

**Conclusions:**

The study results will inform the further implementation and scale-up of CHAMP across primary care centers in Singapore. Successful implementation of digital health interventions to support self-management of chronic disorders may improve health care delivery without further straining health care systems.

**Trial Registration:**

PROSPERO CRD42024613653; https://www.crd.york.ac.uk/PROSPERO/view/CRD42024613653

**International Registered Report Identifier (IRRID):**

DERR1-10.2196/72942

## Introduction

### Background

Hypertension is a leading risk factor for cardiovascular disease worldwide [1]. It is estimated that hypertension affects approximately 31% of the global adult population [2] and a similar proportion of people in Singapore, an island state in Southeast Asia [3]. Despite this high prevalence, approximately half of those affected are unaware of their condition [3,4]. Furthermore, approximately 25% of the people with a known diagnosis of hypertension are not treated, and two-thirds of people receiving treatment are not well controlled [3-5]. Home blood pressure monitoring (HBPM) is an important component of effective hypertension treatment [6-11]. It is associated with improved blood pressure (BP) control [7,10,11] and is cost-effective [12]. However, only 50% of individuals with hypertension adhere to HBPM programs [12,13] due to a variety of reasons, such as the lack of knowledge about the benefits of HBPM; associated costs of acquiring a BP monitor; and low engagement with long-term, rigid HBPM protocols [12-14]. Furthermore, health care providers (HCPs) often report concerns about the accuracy of measurements and devices and also about patient anxiety associated with BP readings that may increase the practice and staff burden [12,14,15].

The effectiveness of HBPM appears to increase if cointerventions are implemented. A systematic review conducted in 2017 reported a pooled decrease in systolic BP from –1.0 mm Hg (95% CI –3.3 mm Hg to 1.2 mm Hg) with no cointervention support to –6.1 mm Hg (95% CI –9.0 mm Hg to –3.2 mm Hg) when personalized support was provided either face-to-face or via teleconsultations [11]. The use of digital interventions in health care settings is increasing, and it has the potential to enhance health care delivery while decreasing costs. Digital health interventions, delivered using mobile devices (apps and other mobile health [mHealth] interventions) or virtual consultations with HCPs, are effective in decreasing systolic and diastolic BP in patients with hypertension [16,17]. Particularly, multimodal interventions, including medication and BP monitoring reminders, health education, and healthy lifestyle advice, appear effective in lowering BP in patients with hypertension [18,19]. Artificial intelligence (AI)–based chatbots have recently emerged as an alternative tool to deliver reminders and other behavioral prompts to support the self-management of chronic disorders by encouraging real-time monitoring, providing patient education and clinical decision support, and offering more personalized services to patients and HCPs [20,21]. Recent systematic reviews offer preliminary evidence that AI-based chatbots promote healthy lifestyle change [22-25]; smoking cessation [26,27]; and self-management of chronic disorders [6,21,28], such as hypertension [29]. In addition, HCPs [30] and patients [31-33] seem to find chatbots acceptable and would be willing to use them, particularly if they complement clinical care [29,33] and are not used for severe conditions [33,34]. However, users have also expressed concerns regarding data privacy and confidentiality and the accuracy of advice provided by the chatbots [30,32].

**Figure 1:**
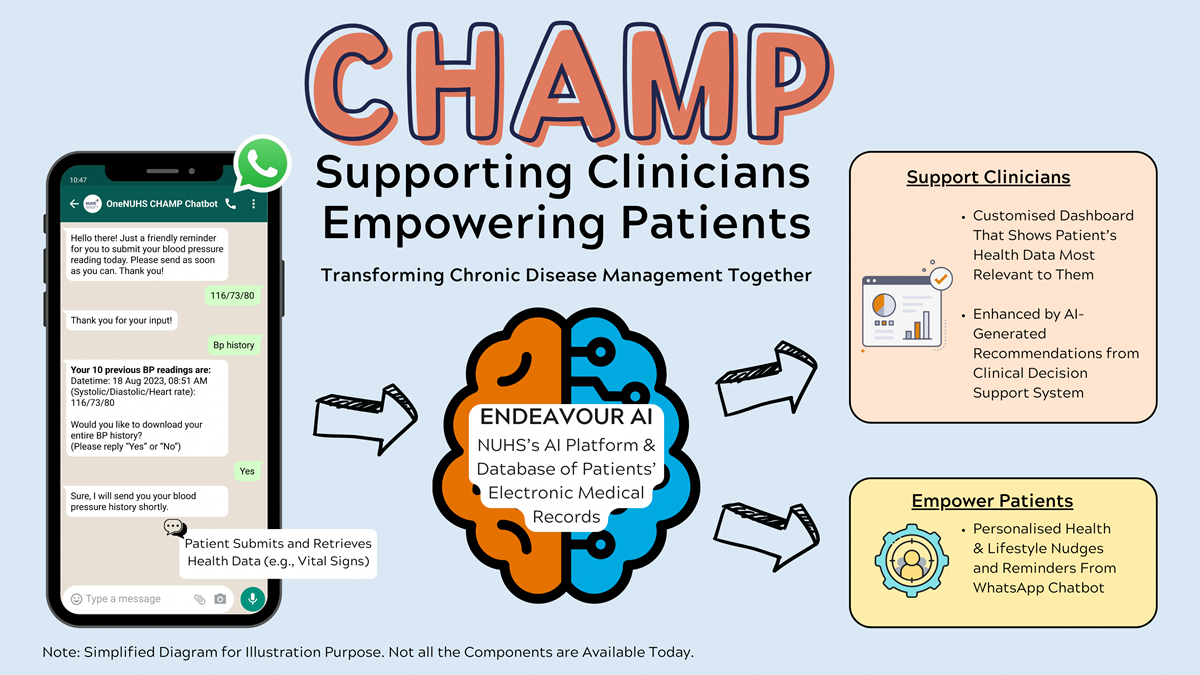
Diagrammatic overview of the Chronic Disease Management Program (CHAMP) chatbot interface and functionalities. Patients enrolled in CHAMP (left) receive regular messages through a WhatsApp chatbot reminding them to monitor their blood pressure (BP) and send the results in a systolic or diastolic BP and heart rate format. The chatbot also advises patients’ next actions according to the submitted BP readings (eg, to repeat the BP reading and to consult with a health care provider). Patient data are received by Endeavor AI (center), the academic health system’s proprietary artificial intelligence (AI) platform. The data collected will feed proprietary models that inform the frequency of patients’ reminders and advice and a customized patient information dashboard for health care providers (right). NUHS: National University Health System.

Chronic Disease Management Program (CHAMP) is a recently developed digital health intervention that aims to provide chronic disease management support to patients and HCPs. The intervention consists of a patient-facing chatbot deployed in WhatsApp (Meta Platforms, Inc), a popular messaging platform, and an AI-augmented clinical decision support system that is linked to electronic medical records (Figure 1). The current version of the chatbot sends regular reminders to patients to measure their BP and share their readings and provides advice on follow-up actions if the BP is outside the reference range for the patient. Future versions of CHAMP aim to include self-monitoring support for diabetes, hyperlipidemia, and other chronic disorders. These reminders will incorporate insights from behavioral science to deliver messages (eg, lifestyle advice) that are meaningful, personalized, and timely to encourage self-monitoring. CHAMP was developed within a large academic health system serving the population of western Singapore, comprising 4 general hospitals and 7 government-funded primary care centers serving a population of approximately 1 million inhabitants. CHAMP is currently being implemented in all primary care centers within the academic health system. However, the implementation and scale-up of CHAMP across several primary care centers requires a systematic, evidence-based approach to ensure that the system is effectively integrated into existing workflows and that patients are able and willing to use CHAMP. There remains a knowledge gap about how best to introduce digital technologies, such as CHAMP, within primary health care services. Earlier studies highlight several potential challenges, such as a lack of initial interest from HCPs, poor integration into current workflows, complex digital interventions, a lack of training, and a lack of leadership support [35-37].

### This Study

This protocol outlines 3 interlinked research studies on the implementation of CHAMP for hypertension self-monitoring in western Singapore to test the overall hypothesis that CHAMP’s deployment is feasible and can be optimized at scale, such that it will benefit primary care center operations by streamlining the management of patients with high BP. This research aims to optimize the deployment of CHAMP across primary care centers in western Singapore by developing effective implementation strategies, informed by the findings of studies 1 and 2. At the same time, this study will use a methodology that is commonly used in implementation studies in Western, high-income country settings but has been used more sparingly in Asian settings. Therefore, this study also intends to test whether a similar methodology is feasible for implementation studies in Asian settings.

## Methods

### Overview

**Figure 2:**
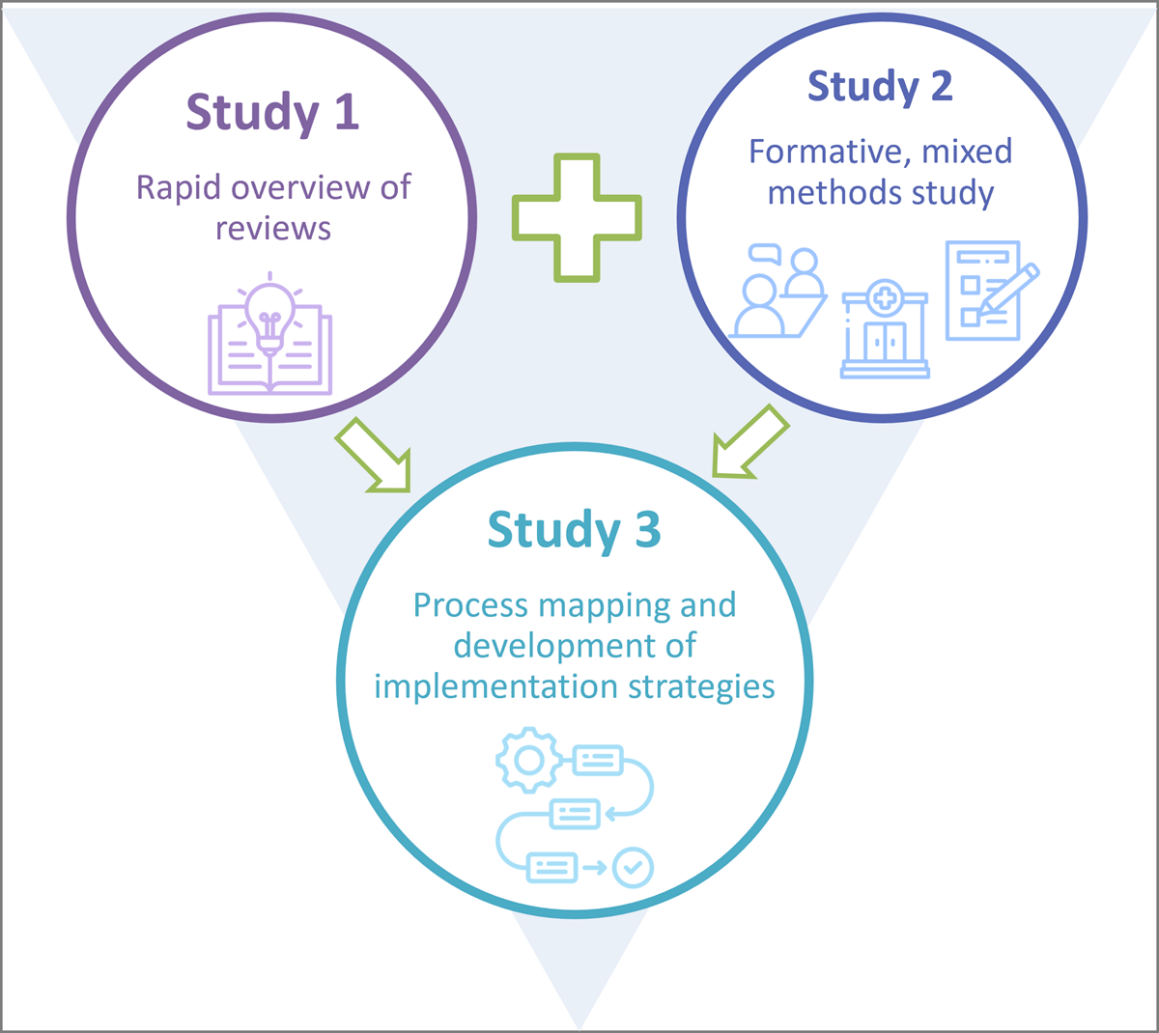
Diagram of the links between the 3 studies presented in this protocol.

This project consists of 3 interlinked implementation studies. Study 1 consists of a rapid umbrella review to describe the global evidence on the factors influencing the implementation of chatbots and other mHealth interventions for chronic disease management and the implementation strategies used to implement these interventions in health care settings. Study 2 will evaluate the barriers and facilitators influencing the implementation of CHAMP in primary care centers in western Singapore by conducting semistructured interviews with relevant stakeholders, assessing the state of CHAMP implementation to date through site visits to all primary care centers and assessing the organizational readiness to implement CHAMP. Finally, study 3 will develop a process map and will generate a set of implementation strategies informed by the results of studies 1 and 2 to enhance the implementation of CHAMP, along with generating a suite of personalized messages to deploy in CHAMP based on evidence-based behavioral science. Figure 2 presents the links between the 3 studies.

### Study 1: Rapid Evidence Review

#### Study Aims

The rapid review aims to assess the factors that influence the implementation of mHealth interventions in health care settings and the implementation strategies and processes used to implement them. Specifically, we will focus on the factors, strategies, and processes influencing the implementation of conversational agents (or chatbots) for chronic disease self-management. The research questions guiding the review are as follows:

1. What are the barriers and facilitators influencing the adoption and implementation of conversational agents (or chatbots) for chronic disease management in health care settings?
2. What implementation strategies are currently used to facilitate the adoption and implementation of the conversational agents (or chatbots) in health care settings?
3. What are the processes used to implement and evaluate these interventions?

#### Methods

The review will follow the Cochrane Rapid Reviews Methods Group [38] methodology. We have registered our review protocol on PROSPERO (CRD42024613653), and we will report our findings according to the PRIOR (Preferred Reporting Items for Overviews of Reviews) statement [39].

#### Search Strategy

We will develop a comprehensive yet focused search strategy combining terms from 3 concepts: conversational agents, implementation strategies, and chronic diseases. The chronic diseases concept will be refined according to the number of retrieved articles to focus only on cardiovascular disease. The search will be restricted to evidence synthesis studies using a validated search filter [40]. Multimedia Appendix 1 provides the MEDLINE search strategy. The searches will be conducted in MEDLINE, Embase, and Web of Science.

#### Eligibility Criteria

##### Overview

This rapid overview or review will include evidence synthesis studies, such as systematic reviews, scoping reviews, and mapping reviews and nonsystematic literature reviews. Articles reporting on primary studies, protocols, editorials, conference abstracts, or letters to the editor will be excluded. We will limit our search to articles in English, published since 2014.

The eligibility criteria will be defined according to the population, intervention, comparison, outcome, and setting (PICOS) framework.

##### Population

We will include reviews reporting on adults (aged ≥21 years) with cardiovascular diseases, such as hypertension, diabetes, and hypercholesterolemia. Reviews reporting on other chronic diseases, such as cancer, mental health disorders, and rheumatological and autoimmune disorders, will be excluded from the overview.

##### Intervention

We will include studies involving mHealth interventions, such as conversational agents (or chatbots) or applications delivered using a mobile digital device such as a smartphone, tablet computer, or wearable device. Nondigital interventions and other digital interventions, such as teleconsultations, passive collection of sensor data, or interventions targeting health care providers or the automation of health care processes, will be excluded from the review.

##### Outcomes

The included studies will report any of the following outcomes: (1) barriers and facilitators influencing the implementation of conversational agents, (2) implementation strategies used in the implementation of conversational agents, and (3) processes and process adaptations leading to the implementation of conversational agents.

##### Setting

We will include studies describing interventions delivered in any health care setting, including community or primary health care facilities, the outpatient clinics of general or specialist hospitals, and secondary and tertiary health care institutions.

#### Data Collection and Analysis

The results of the database searches will be uploaded to EndNote (version 21; Clarivate), and duplicate citations will be removed. The resulting library will be uploaded to Rayyan (Rayyan Systems, Inc), a web-based tool developed to facilitate screening of articles for systematic reviews [41]. The screening process will include 2 phases: title and abstract screening and full-text screening. Following a rapid review methodology, the screening will be initially conducted by 2 reviewers working in parallel (10%-20% of the library) to standardize the screening process and agree upon eligibility criteria. Subsequent screening will be conducted by 1 reviewer. Disagreements that arise during screening will be resolved through discussion between the reviewers or by engaging a third reviewer. The study selection process will be summarized in a PRISMA (Preferred Reporting Items for Systematic Reviews and Meta-Analyses) flow diagram [42].

**Figure 3:**
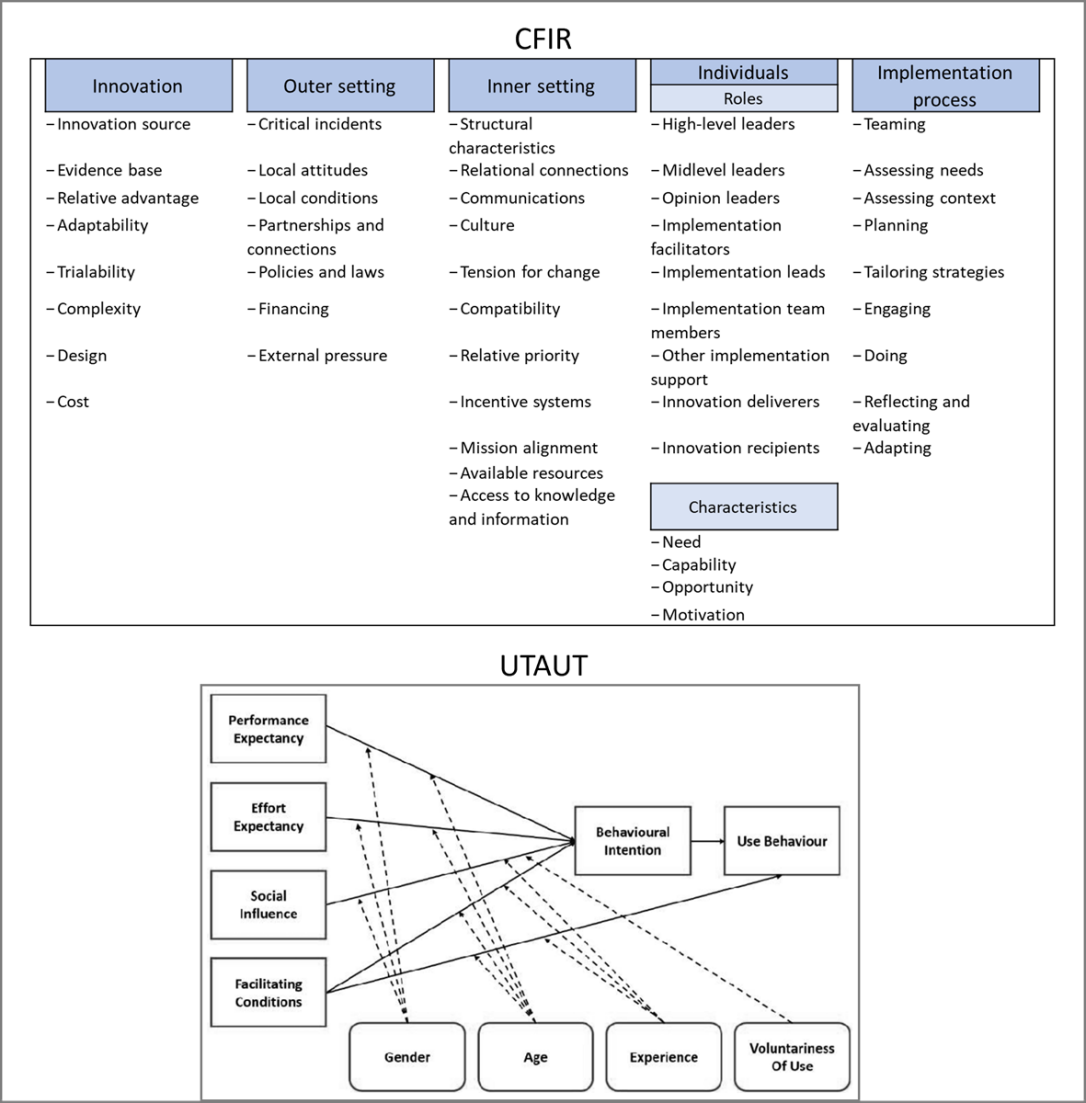
Components of the Consolidated Framework for Implementation Research (CFIR) and the unified theory of acceptance and use of technology (UTAUT) frameworks to be used to code retrieved evidence in study 1.

Data from the included studies will be extracted in a Microsoft Excel form developed by the research team. Two reviewers, working independently and in parallel, will extract data for about 10% of the included studies to pilot the data extraction form and standardize data extraction. Subsequently, data extraction will be performed by 1 reviewer. The data extraction form will be informed by the Consolidated Framework for Implementation Research (CFIR) [43,44] and the unified theory of acceptance and use of technology (UTAUT) [45] frameworks. CFIR [44] is a widely used framework in implementation science to determine the contextual factors influencing implementation. UTAUT [45] is an encompassing framework widely used in digital health to evaluate the factors influencing the adoption of digital health interventions. As CFIR is a generic implementation framework, we will use the UTAUT framework to complement the “innovation” and “individuals” sections of CFIR, with a framework focusing specifically on the acceptance of digital health interventions. Figure 3 [45] presents the components of each framework.

Data analysis will follow the framework analysis methodology [46]. We will use CFIR and UTAUT to develop a framework that will guide data coding. We will follow a deductive coding approach for data analysis. Two reviewers (LM and NHLH) will perform the data analysis independently and in parallel. Reviewers will meet regularly to review their findings and discuss any disagreements. If the reviewers are unable to reach a consensus, a third reviewer (last author) will act as an arbiter.

#### Expected Outcomes

The overview will provide global evidence on the barriers and facilitators influencing the implementation of mHealth interventions (including chatbots) and the implementation strategies and processes used when implementing these interventions. The research data from the review will be made available through a peer-reviewed publication.

### Study 2: Formative Mixed Methods Study

#### Study Aims

Study 2 aims to assess the state of CHAMP deployment to date, the organizational structure of the primary care centers implementing CHAMP, and the perceived barriers and facilitators of the implementation and scale-up of CHAMP. We will address the following research questions:

1. What are the barriers and facilitators influencing the adoption and implementation of CHAMP in the primary care centers where the chatbot has been implemented at the time of the study?
2. What is the readiness to change among HCPs and leadership teams at the primary care centers?
3. What are the processes and implementation strategies used in the primary care centers to implement CHAMP to date?
4. What are the most effective and locally appropriate strategies used to implement CHAMP in the primary care centers?

#### Methods

##### Overview

Study 2 will include a mixed methods study comprising 2 qualitative, complementary methods: semistructured interviews with relevant stakeholders and 1-day site visits to carry out on-site observation and a quantitative survey to assess readiness to change. This study requires ethics approval from 2 boards. It is currently under review by Singapore’s Ethics and Compliance Online System and has been reviewed (and approved) by the National University of Singapore Institutional Review Board (NUS-IRB-2024-669). The findings from study 2, taken together with the results from study 1, will be used to develop an implementation process map and design relevant implementation strategies in study 3.

##### Semistructured Interviews

###### Study Population

We aim to interview the following stakeholders:

- Members of the CHAMP development and initial implementation team who were involved in the initial deployment of CHAMP in primary care centers
- Primary care centers’ leadership
- HCPs, including family physicians and care managers (nurses trained to provide follow-up care and health education to patients with chronic conditions), who are participating and those who are not participating in CHAMP’s rollout
- Adult patients (aged ≥21 years) with a clinical diagnosis of hypertension or who require frequent BP monitoring for other conditions, who are enrolled or not enrolled in CHAMP

###### Sample Size

We aim to recruit between 40 and 60 participants for the interviews. We will use purposive sampling, recruiting 15 to 30 HCPs and 15 to 30 patients from the 7 primary care centers, 3 to 5 members of the primary care centers’ leadership, and the members of the CHAMP development and initial implementation team. The total number of interviewees will be determined by the eligible population (number of HCPs, leaders, and members of the CHAMP team eligible and willing to participate in the study) and, particularly for patients, by reaching thematic saturation [47-49], defined as the number of interviews when no new codes emerge from the data. This was estimated to be approximately 12 interviews by Guest et al [49].

###### Participant Recruitment

We will use different recruitment modalities according to the participants’ group.

First, the members of the CHAMP development and initial implementation team, HCPs, and primary care centers’ leadership will be invited to participate in the interview via email. The place, time, and modality (face-to-face or virtual) of the interview will be agreed with the participant before the interview. We will aim to interview HCPs involved and not involved in patient recruitment for CHAMP.

Second, the patients currently enrolled in CHAMP will receive a message via CHAMP’s WhatsApp channel, enquiring their willingness to participate in research associated with CHAMP.

Third, the patients who are not enrolled in CHAMP will be recruited face-to-face during their regular check-up visits at the primary care center.

###### Procedure

We will conduct one-to-one semistructured interviews following an interview guide. Interviews are estimated to last 30 to 45 minutes and will be digitally recorded for subsequent analysis. The interviews will have a retrospective focus to understand CHAMP’s implementation process to date, lessons learnt, and the factors that influenced its implementation.

The interviews will be developed by the research team and will be based on the CFIR [44] and the UTAUT [45] frameworks (Multimedia Appendix 2). The interview guides will be piloted with 1 or 2 participants to ensure content and context relevance, feasibility, and a suitable flow of questions. We will use the pilot participants’ feedback to edit the interview guides before starting data collection.

###### Data Analysis

Recordings of the interviews will be transcribed verbatim. The interview data will be analyzed using the framework analysis approach as outlined by Gale et al [46]. The analysis will be guided by the CFIR [44] and UTAUT [45] domains. Two researchers working independently and in parallel will deductively code the data using codes developed in advance by the research team. If the interviews include relevant information that would not fit the codebook, the researchers will inductively develop new codes. The research team will code the first 3 transcripts in parallel, following which they will meet to discuss the coding and reach consensus on the final codes and themes. Subsequently, the researchers will code the rest of the transcripts independently. During the whole process, the research team will meet, discuss, and reach consensus on the final codes and themes. We will use NVivo (version 14.0; Lumivero) to code the data.

##### One-Day Site Visit

###### Overview

We will conduct a series of observations in the primary care centers to assess their structure, staff-to-patient ratio, demographic of the patient population, patient journey, the number of research or implementation projects the primary care institution is currently involved with, and other relevant information. Concurrently, we will aim to understand CHAMP’s current implementation workflow. This includes the number of HCPs involved in CHAMP’s implementation, steps to enroll patients, type of HCP (physician and care manager) enrolling and educating patients on the use of CHAMP, duration of enrollment or education procedure, and the provision of written materials to reinforce learning. If possible, the site visit will include the observation of “live” patient enrollment into CHAMP.

###### Procedure

One-day visits to all primary care centers in western Singapore will be conducted by 2 to 3 members of the research team. The date and time of the visit will be arranged with the primary care center leadership. During the visit, the researchers will passively observe the primary care center’s operations and, if required, engage in informal interviews with the leadership team, administrators, and HCPs to complement the observations. We will attempt to observe clinical consultations by physicians and care managers, if possible. Findings from the observations and informal interviews will be recorded in a structured form developed by the research team. Verbal consent will be taken before the stakeholder informal interviews.

##### Readiness to Change Assessment

###### Study Population

We will include the following stakeholders: leadership teams and HCPs, including physicians and care managers working in chronic disease management teams in primary care centers. We will invite all eligible HCPs to participate in the study and aim to collect responses from at least 50% of the eligible population.

###### Study Design

Readiness to change will be assessed using 2 brief self-reported questionnaires.

First, the Organizational Readiness for Implementing Change (ORIC) [50] is a reliable and validated questionnaire that evaluates readiness to change at an organizational level by assessing the concepts of change commitment (eg, “people who work here are determined to implement CHAMP”) and change efficacy. This measure is based on the theory of the determinants and outcomes of organizational readiness for change by Weiner [51] and consists of 12 statements rated using 5-point Likert scales.

Second, the Readiness Thinking Tool (or Readiness Monitoring Tool) [52,53] assesses readiness at an organizational level. This validated tool contains 19 elements grouped in 3 constructs: motivation (6 elements), innovation-specific capacity (5 elements), and general capacity (8 elements)—all rated on 4-point Likert scales. Multimedia Appendix 3 presents the ORIC [50] and Readiness Thinking Tool [52,53] surveys.

###### Participant Recruitment and Study Procedure

Participants will be invited to participate in the study via email. The invitation email will contain a link to the surveys for the participants to access if they agree to be part of the study.

###### Data Analysis

We will use descriptive statistics to analyze the data. We will perform subgroup analyses to determine the readiness to change among specific HCP groups (eg, physicians, care managers, and nurses) and primary care center leadership teams and HCPs working in each of the primary care centers included in the study. The results of these assessments will offer a perspective on the implementation context for CHAMP across sites and will be used to design site-specific targeted implementation strategies to optimize the implementation and scale-up of CHAMP.

#### Expected Outcomes

Study 2 will produce a comprehensive, contextually relevant list of barriers and facilitators of the implementation of CHAMP in primary care centers in western Singapore, including the HCPs’ readiness to implement CHAMP.

### Study 3: Development of Implementation Strategies for the Implementation of CHAMP in Primary Care Centers

#### Study Aims

In study 3, we aim to develop a set of implementation strategies to effectively implement CHAMP and evaluate the outcomes of implementation in the primary care centers. We will also use behavioral science frameworks to enhance patients’ engagement with CHAMP. The research questions guiding this study are as follows:

1. What are the most appropriate and effective strategies to implement CHAMP in the primary care centers of western Singapore?
2. How and what aspects of CHAMP can be improved to increase patients’ engagement with the platform and, ultimately, more positive health outcomes?

#### Methods

##### Process Mapping

The information obtained in study 1 (rapid evidence review) and study 2 (on-site visits, semistructured interviews, and readiness to change surveys) will be used to develop a process map to outline the patient journey and map the barriers and facilitators influencing the current deployment of CHAMP. We will use the implementation research logic model [54] as a template to outline the process map. Once completed, the process map will be shared with relevant stakeholders (analogous to the groups from which study 2 interview participants are drawn) for input and further refinement.

The iterative development of the process map will assist the research team in outlining the implementation strategies, mapping these strategies to the relevant influencing factors, and the proposed theories of change and mechanisms of implementation. The process map will be revisited at regular intervals during study 3 (see the Development of Implementation Strategies section) to evaluate the progress and success of CHAMP implementation. To evaluate the implementation success, we will attempt (in future studies) to compare the intended outcomes of implementation, as outlined in the process map, with the actual results of implementation and how stakeholders evaluate the outcomes and success of this exercise.

##### Development of Implementation Strategies

The findings from studies 1 and 2, coupled with the readiness assessments mentioned earlier, will inform the development of implementation strategies to enhance CHAMP’s implementation and scale-up. We will develop a set of evidence-informed, pragmatic, context-driven implementation strategies to address the barriers to and enhance the facilitators of implementation. We will use the Expert Recommendations for Implementing Change taxonomy [55] to define the implementation strategies, and the action, actor, context, target, and time framework [56] to define the strategy implementation processes. The implementation strategies will be developed by the research team in collaboration with the CHAMP development and implementation team and the HCPs in the primary care centers. Examples of implementation strategies and how they could address specific barriers are shown in Table 1. The implementation strategies will be presented in the form of a pragmatic research logic model booklet and will be used to guide subsequent implementation and scale-up of CHAMP.

**Table 1:** Examples of barriers that are associated with implementation strategies based on the Expert Recommendations for Implementing Change (ERIC) and action, actor, context, target, and time (AACTT) frameworks.

Targeted barrier	ERIC strategy	Actor	Action	Context	Target	Time
Lack of a clear directive from the top management	Mandate use	Head of the department	Mandates the use of CHAMP^a^	Primary care center	HCPs^b^ who will prescribe CHAMP	At the department meeting
The clinical team not familiar with CHAMP	Conduct ongoing training	CHAMP team	Conducts workshop on CHAMP functionalities	Primary care center	HCPs who will prescribe CHAMP	Before launching the implementation study
Patients unsure about enrollment	Involve patients	HCPs who will prescribe CHAMP	Empower and activate patients to use CHAMP	Primary care center	Patients	During clinical consultation
Patients who miss submitting blood pressure readings	Intervene with patients to enhance adherence	HCPs who will prescribe CHAMP	Embed and test behavioral levers	Reminder messages	Patients	During the stipulated self-monitoring period

^a^CHAMP: Chronic Disease Management Program.

^b^HCP: health care provider.

##### Leveraging Behavioral Science

###### Overview

Effective implementation of evidence-based practice depends on behavior change. In relation to CHAMP, a key indicator of success rests on patients’ interactions with the chatbot and compliance with the messages (eg, reminders to submit BP readings and lifestyle advice) it conveys. To enable this, researchers have developed numerous frameworks for guiding the development, design, and implementation of behavior change interventions, one of which features prominently in the digital health literature—MINDSPACE (messenger, incentives, norms, defaults, salience, priming, affect, commitments, and ego) [57]. The MINDSPACE framework represents 9 different evidence-based strategies for promoting behavior change in people. These strategies have been used in a variety of health situations, ranging from the promotion of vaccination uptake, regular exercise, and healthy eating habits to smoking and alcohol cessation [57]. Unlike traditional cognitive models that assume people consciously analyze their options and act in ways that are consistent with their own best interests, contextual models, such as MINDSPACE, tend to focus on people’s reliance on automatic processes of judgment to make decisions [58]. Hence, while cognitive strategies emphasize reflection, training, persuasion, and education, MINDSPACE leverages aspects of the choice or contextual environment to bring about behavior change in people. A recent meta-analysis of 14 digital intervention studies relying on different MINDSPACE strategies showed that digital interventions improved cancer screening uptake and adherence among at-risk individuals [59]. Digital interventions relying on MINDSPACE hold considerable promise in promoting behavior change in a relatively cheap, quick, and efficient manner. Drawing on the findings of studies 1 and 2, we will use MINDSPACE to systematically test variations of the chatbot’s messages in effectively engaging patients. A meta-analysis of 40 web-delivered health behavior change programs has shown that tailored interventions (eg, personalization, interactivity, frequency, and convenience) resulted in significantly better health outcomes compared with control conditions at posttest and follow-up time points [60]. In CHAMP, one approach could be the use of behavior-informed messages to increase physical exercise in patients.

###### Study Design

Study 3 uses a randomized controlled trial design to evaluate the effectiveness of behavior-informed messages (administered via CHAMP) in promoting physical exercise among patients. The intervention compares the current practice of sending standard education messages about the benefits of exercise (ie, control) with behavioral-informed messages that encourage physical activity in patients through goal setting (ie, intervention group) as well as goal setting and feedback (ie, intervention plus group).

###### Procedure

Patients will be randomly allocated to 1 of the 3 study arms using a computer-generated randomization sequence created via Python’s (Python Software Foundation) random module. The allocation sequence will be concealed from the study team before assignment to minimize selection bias. Randomization will occur at the individual patient level. Participants will be assigned to 1 of the 3 groups mentioned subsequently.

The first group is the control group. This group represents the current status quo in which patients receive regular information about the benefits of exercise.

The second group is the intervention group. In addition to regular information about the benefits of exercise, patients in this group will receive regular prompts to log their exercise activity in the past week and their commitment in the following week. *Commitment* is one of several strategies in MINDSPACE.

The third group is the intervention plus group. In addition to regular information about the benefits of exercise, patients in this group will receive regular prompts to log their exercise activity in the past week and their commitment in the following week. They will also receive regular feedback on how often or well they are doing this (eg, frequency and adherence) relative to their peers to demonstrate social norms of physical activity. *Commitment* and *norms* are 2 of several strategies in MINDSPACE.

All messages will be delivered through the official CHAMP WhatsApp chatbot. Messaging timing and frequency are consistent across all groups.

###### Participants and Recruitment

Participants will be recruited from a pool of patients who are currently enrolled in the CHAMP system, an active platform using WhatsApp to send health-related prompts for BP monitoring. At the end of the initial 6-month data collection period, a data analyst will extract and anonymize the relevant data from the CHAMP system. The study team will only receive anonymized data to assess the outcomes.

###### Outcomes

Objective indicators: pre-and post-intervention’s weight and BP levels will be extracted from health records data obtained during routine clinic visits.

Physical activity in the past 6 months: we will use a single self-reported question assessing patients’ level of physical activity in the past 6 months. The questionnaire will be administered as part of routine care in the polyclinics, pre- and postintervention.

### Data Analysis

We will perform a series of regressions to analyze our outcomes of interest. We will treat the intervention groups as treatment variables and use the current education-only group as the reference group. Hence, 1 of the aims of study 3 is to improve the chatbot’s ability to engage with patients and, through this, improve patients’ health outcomes. This not only supports evidence-based practice but also highlights the value of CHAMP to clinicians, patients, and the community at large.

### Expected Outcomes

Study 3 will use data triangulation from the rapid overview of reviews (study 1), on-site visits, semistructured interviews, and readiness to change surveys (study 2) to inform the development of a process map summarizing the patient journey and factors influencing CHAMP implementation. The process map will inform the development of implementation strategies to enhance the current implementation of CHAMP in primary care centers. Finally, we will work with the CHAMP development team to enhance and personalize the chatbot’s messages to patients, leveraging behavioral science and relevant findings from studies 1 and 2.

### Ethical Considerations

Study 1 is a review of published literature and does not require ethics approval. For study 2, interviews with the CHAMP development and initial implementation team, HCPs, and primary care centers’ leadership have been approved by the National University of Singapore Institutional Review Board (NUS-IRB-2024-669). For patients’ interviews and on-site visits, we are awaiting approval by Singapore’s Ethics and Compliance Online System. All participants will sign an informed consent form before the start of the studies (semistructured interviews and readiness to change surveys) and will be compensated for their participation in the studies. Any personal identifiable data will be stored in secure university servers. Only deidentified participant data will be used in papers or other dissemination materials.

### Dissemination of the Study Results

Findings from these studies will be shared with the funders and stakeholders involved in CHAMP’s development, including patients using the platform. Research findings will be presented at conferences and published in relevant peer-reviewed journals.

## Results

To date, the following aspects of the studies have been initiated. For study 1, we registered the protocol on PROSPERO (CRD42024613653), developed the search strategy, and conducted the search on October 4, 2024, in MEDLINE, Embase, and Web of Science, retrieving 2835 citations. After deduplicating the library, we completed title and abstract screening and are currently conducting the full-text screening. For study 2, we obtained ethics approval for the interviews to HCPs, primary care centers’ leadership, and CHAMP development and initial implementation teams. We completed 31 semistructured interviews. We obtained ethics approval for patient interviews and on-site visits, and they are currently ongoing. We started administering the readiness to change survey on February 3, 2025, and data collection is ongoing.

## Discussion

### Anticipated Findings

This paper presents a protocol for a multimethod, multistage study, aiming to implement a digital health intervention to support chronic disease self-management, such as hypertension, in government-funded primary care centers in western Singapore. Hypertension remains an important risk factor for cardiovascular disease and incurs the highest societal cost of modifiable risk factors, including metabolic, lifestyle, environmental, and substance use risk factors [61]. This study is among the first in Asia to assess and enhance the implementation of an AI-based digital intervention in primary care. The results from this research will inform the further implementation and scale-up of CHAMP across primary care centers in Singapore. Furthermore, this study will provide relevant contextual information to better design targeted implementation strategies to enhance the facilitators and overcome the current barriers to the implementation of CHAMP. The successful implementation of digital health interventions to support the self-management of chronic disorders, such as hypertension, may improve health care delivery without further straining health care systems.

### Strengths and Limitations

An important strength of this protocol is presenting a multimethod, multistage study that will apply widely used, evidence-based implementation science frameworks and methodologies to design contextualized implementation strategies. The research presented in this protocol integrates frameworks specifically developed for use with digital interventions alongside well-established implementation frameworks. Applying them concurrently and evaluating their relevance and usefulness in driving the research and its reporting will offer a case study for how implementation science studies with a similar focus and research questions might be conducted. The rapid expansion of digitally delivered and AI-driven interventions and technologies in the last few years suggests that studies with such a focus will proliferate in the future. Limitations of this project include the implementation in primary care centers whose structure is unique to the Singapore context and the multicultural mix of the Singaporean population from which study participants will be drawn. Consequently, the findings of this study may not be fully transferable to other health care systems and populations.

### Dissemination Plan

The findings from this study will be published in at least 1 peer-reviewed journal article. We also aim to present the studies’ results in relevant scientific meetings or conferences. Beyond scientific dissemination, we will also undertake knowledge mobilization activities within the study context. These will include targeted events with study stakeholders and their peers, including those from other health care clusters and geographies in Singapore. We envisage preparing evidence briefs and hosting facilitated sessions (eg, workshops and webinars), such that policy and clinical stakeholders can identify relevant knowledge and data from this research that are suitable to inform their implementation planning within the domain of hypertension management and other chronic diseases.

## Appendix

Multimedia Appendix 1 Search strategy—MEDLINE (October 4, 2024).

Multimedia Appendix 2 Semistructured interview guides.

Multimedia Appendix 3 Organizational Readiness for Implementing Change and Readiness Thinking Tool surveys.

## Abbreviations

AI: artificial intelligence
BP: blood pressure
CFIR: Consolidated Framework for Implementation Research
CHAMP: Chronic Disease Management Program
HBPM: home blood pressure monitoring
HCP: health care provider
mHealth: mobile health
MINDSPACE: messenger, incentives, norms, defaults, salience, priming, affect, commitments, and ego
ORIC: Organizational Readiness for Implementing Change
PICOS: population, intervention, comparison, outcome, and setting
PRIOR: Preferred Reporting Items for Overviews of Reviews
PRISMA: Preferred Reporting Items for Systematic Reviews and Meta-Analyses
UTAUT: unified theory of acceptance and use of technology
