# Supplementary material for: Implementation of a Digital Health Intervention (CHAMP) for Self-Monitoring of Hypertension: Protocol for 3 Interlinked Implementation Studies
Source: JMIR Res Protoc. 2025 Oct 17;14:e72942. doi: 10.2196/72942 (PMC12579285; doi:10.2196/72942)
Supplement: Multimedia Appendix 1 [file resprot_v14i1e72942_app1.docx]

## Appendix 1

### Search strategy – MEDLINE (4 October 2024)

#### Concept 1_ mHealth & chatbots

(Title/Abstract/Keywords) mobile app OR mobile apps OR smartphone app OR smartphone apps OR health app OR health apps OR mHealth app OR mHealth apps OR eHealth app OR eHealth apps OR mobile application* OR mobile intervention* OR mobile based OR cell phone OR smart phone OR smartphone OR mobile health OR mHealth OR chatbot OR chat bot OR conversational agent OR conversational avatar OR conversational bot OR conversational AI OR dialog* system OR embodied agent OR intelligent agent OR intelligent assistant OR relational agent OR virtual assistant OR virtual agent OR virtual advisor OR virtual coach OR computer agent

AND

#### Concept 2_Implementation

(MeSH) Implementation Science OR

(Title/Abstract/Keywords) implement* OR adopt* OR uptake OR Appl* OR Carry-out OR Perform* OR Usage OR Practice OR Enactment OR Fulfil* OR knowledge-transfer* OR feasib* OR adapt* OR accept* OR appropriate* OR cost* OR fidelit* OR sustainab* OR penetrat* OR reach* OR utili* OR sustained-integration* OR intention-to-chang* OR embed* OR normali* OR Disseminat* OR diffus* OR (translat* adj3 knowledge*) OR scale-up OR scaling OR barrier* OR change-in-practice* OR Obstruct* OR Obstacle* OR Facilitat* OR Threat* OR factor*OR access* OR retention OR retain* OR compatib* or brakes OR levers OR (perception* adj3 attitude*) OR evaluat* OR roll-out

AND

#### Concept 3_Chronic disease

(MeSH) acute coronary syndrome OR cardiovascular diseases OR coronary disease OR coronary artery disease OR diabetes mellitus OR diabetes mellitus, type 2 OR hyperlipidemia OR dyslipidemia OR hypercholesterolemia OR hypertriglyceridemia OR hypertension OR arteriosclerosis OR myocardial ischemia

(Title/Abstract/Keywords) acute coronary syndrome OR cardiovascular disease* OR coronary disease* OR coronary heart disease* OR coronary artery disease* OR diabetes OR type 2 diabetes OR hyperlipidemia OR hyperlipidaemia OR lipidemia OR lipidaemia OR high cholesterol OR dyslipidemia OR dyslipidaemia OR hypercholesterolemia OR hypercholesterolaemia OR hypertriglyceridemia OR hypertriglyceridaemia OR high blood lipid* OR hypertension OR high blood pressure OR arteriosclerosis OR atherosclerosis OR myocardial ischemia

AND

#### Concept 4_Publication type

(Publication type) meta-analysis OR review OR

(MeSH) meta analysis OR

meta analysis OR search*
