# Supplementary material for: Implementation of a Digital Health Intervention (CHAMP) for Self-Monitoring of Hypertension: Protocol for 3 Interlinked Implementation Studies
Source: JMIR Res Protoc. 2025 Oct 17;14:e72942. doi: 10.2196/72942 (PMC12579285; doi:10.2196/72942)
Supplement: Multimedia Appendix 2 [file resprot_v14i1e72942_app2.docx]

## Appendix 2

### Semi-structured interview guides

**Healthcare providers**

Thank you so much for contributing your time to be part of our discussion today.

You are invited to this interview because you are a member of the healthcare team providing patient care for chronic medical conditions.

We are conducting an implementation study to improve the uptake and the experience of using CHAMP. You may or may not be involved in CHAMP directly. Through this interview, we would like to understand your opinions about using CHAMP in NUP and how to improve the roll out of CHAMP to better support you and your patients.

The interview will last about 30-45 minutes, but no longer than 60 minutes. It will be audio and video recorded, but only the audio recording will be saved for transcription. Your views will remain anonymous in the transcription, analysis, and publication of the research results.

**BACKGROUND INFORMATION**

1. What is your professional background?
2. How long have you been working in this polyclinic? And in this teamlet?
3. Could you describe a typical day at work?
4. How many patients do you see in a day?

**CHAMP IMPLEMENTATION**

1. Have you used CHAMP to manage your patients before?
   1. (FOR PROVIDERS **NOT** ON CHAMP) Was this a personal decision or mandated by the polyclinic leadership?
   2. (FOR PROVIDERS **NOT** ON CHAMP) (If personal decision) What factors influenced your decision of not offering CHAMP to your patients?
2. (FOR PROVIDERS ON CHAMP) How did you get involved with CHAMP?
3. (FOR PROVIDERS ON CHAMP) What factors influenced your decision of offering CHAMP to your patients?
4. (FOR PROVIDERS ON CHAMP) How would you describe your experience of using CHAMP so far?
   1. In what way do you find CHAMP useful, and not useful?
   2. What features do you find useful? What features could be better?
5. (FOR PROVIDERS ON CHAMP) What are your current roles and responsibilities in this polyclinic/teamlet related to CHAMP?
6. What are your views about introducing CHAMP in the polyclinic?
   1. How does CHAMP impact the current polyclinic workflows?
      1. How beneficial would CHAMP be to improve patient outcomes?
      2. How beneficial would CHAMP be to decrease HCP workload?
      3. How beneficial would CHAMP be to your experience in consults with hypertensive patients?
   2. To what extent is CHAMP compatible with the current polyclinic workflows?
   3. What changes do you think would be required to include CHAMP in the current polyclinic workflows?
   4. To what extent would these changes affect your workload?
7. To what extent is the polyclinic current infrastructure adequate for CHAMP implementation?
   1. What about the physical infrastructure? (polyclinic layout, clinic room organizations, etc)
   2. What about the IT infrastructure?
   3. (If no) Are the required changes feasible?
8. (FOR PROVIDERS ON CHAMP) Could you please describe the process of implementing CHAMP in the polyclinic?
   1. Did you receive training and support from the CHAMP PMO (Project Management Team) before starting using CHAMP?
      1. Was it easy/difficult to learn about CHAMP?
      2. Did you feel supported in the learning/training process?
      3. To what extent did (would) the training and support offered by the CHAMP PMO influence your decision of using CHAMP?
9. (FOR PROVIDERS **NOT** ON CHAMP) Are you aware of what was the process of implementing CHAMP in the polyclinic?
   1. Did you participate in training and support sessions delivered by ~~from~~ the CHAMP PMO (Project Management Team) before CHAMP was rolled out in the polyclinic?
      1. Was it easy/difficult to learn about CHAMP?
      2. Did you feel supported in the learning/training process?
      3. [To ask if participant chose to not be part of CHAMP] To what extent did the training and support offered by the CHAMP PMO influence your decision of using CHAMP?
10. How would you describe the polyclinic leadership support in implementing CHAMP?
    1. To what extent leadership mandates influenced (may influence) this decision?
11. How would you describe the support for CHAMP among your colleagues?
    1. To what extent did (do) your peers’ attitude about using CHAMP influence your decision
12. Are you aware of other interventions similar to CHAMP?
    1. Are they currently been offered to patients?
    2. How does CHAMP compare to those interventions?
       1. Is CHAMP easier/more difficult to use/implement than those interventions?
    3. Are there any advantages or disadvantages of using CHAMP over other interventions?
    4. Are there any advantages or disadvantages of using CHAMP compared to patients not using any blood pressure monitoring system?

**PATIENT RECRUITMENT**

1. What do you think are the needs of polyclinic patients with regards to hypertension care?
2. To what extent do you think CHAMP addresses the patients’ needs?
3. (FOR PROVIDERS ON CHAMP) How are patients recruited to CHAMP?
   1. Are you aware of any difficulties with the recruitment of patients to CHAMP?
   2. Do patients submit the blood pressure readings for 30 consecutive days?
      1. If not, are there any specific actions to nudge patients to do it?
4. (FOR PROVIDERS ON CHAMP) In your experience, how long do patients continue using CHAMP after they have been recruited onto it?
   1. Why do you think patients continue using CHAMP?
   2. What would help them to continue using CHAMP?
5. (FOR PROVIDERS ON CHAMP) What is patients’ feedback about using CHAMP?
   1. What difficulties do they have?

**FINAL THOUGHTS**

1. What do you think of the current implementation of CHAMP overall?
2. What would you do differently if you were to lead the CHAMP implementation process

**CHAMP development and implementation team**

Thank you so much for contributing your time to be part of our discussion today.

You are invited to this interview because you are a member of the CHAMP development team.

We are conducting a study to understand the implementation of CHAMP so far. Through this interview, we would like to understand your involvement in the CHAMP development team and the roll out of CHAMP to date, and how to improve the future implementation of CHAMP further.

The interview will last about 30-45 minutes, but no longer than 60 minutes. It will be audio and video recorded, but only the audio recording will be saved for transcription. Your views will remain anonymous in the transcription, analysis, and publication of the research results.

1. What is your professional background?
2. How long have you been working in the CHAMP development team?
3. Could you describe a typical day at work?

**CHAMP IMPLEMENTATION**

1. How did you get involved with the CHAMP research team?
2. What are your current roles and responsibilities in the CHAMP research team?
3. What are your views about ~~i~~ntroducing CHAMP in polyclinics?
   1. To what extent do you think CHAMP (will) impact(s) the current polyclinic workflows?
      1. To what extent would you think that CHAMP (will) improve(s) patient outcomes?
      2. To what extent would you think that CHAMP (will) decrease(s) HCP workload?
   2. To what extent do you think CHAMP is compatible with the current polyclinic workflows?
      1. What changes do you think would be required to include CHAMP in the current polyclinic workflows?
      2. To what extent do you think these changes will affect the HCPs workload?
4. To what extent is the polyclinic current infrastructure adequate for CHAMP implementation?
   1. What about the physical infrastructure? (polyclinic layout, clinic room organizations, etc)
   2. What about the IT infrastructure?
   3. (If no) Are the required changes feasible?
5. Could you please describe the actions taken by the team to implement CHAMP in the polyclinics?
6. Have you participated in the design or delivery of the training workshops for polyclinic staff conducted ahead of CHAMP implementation?

If yes:

- 1. What was your experience conducting (participating in) the training workshops?
  2. Were HCPs familiar with the concepts and pathways presented in the workshops?
  3. Were there any particular topics that were more difficult to understand?
  4. Did the HCPs expressed that some implementation pathways were difficult to implement given the current polyclinic workflows?

If no, please move to the next question

1. How would you describe the polyclinic leadership support in implementing CHAMP?
   1. To what extent leadership mandates influenced the implementation of CHAMP in the polyclinics?
2. How would you describe the HCPs support for CHAMP?
3. Are you aware of other interventions similar to CHAMP currently offered in the polyclinics?
   1. How does CHAMP compare to those interventions?
      1. Is CHAMP easier/more difficult to use/implement than those interventions?
   2. Are there any advantages or disadvantages of using CHAMP over alternative interventions?
   3. To what extent do you think these interventions influence the willingness of the polyclinics leadership and staff to offer CHAMP?

**PATIENT RECRUITMENT**

1. What do you think are the needs of polyclinic patients with regards to hypertension care?
2. To what extent do you think CHAMP addresses the patients’ needs?
3. How are patients recruited to CHAMP?
   1. Are you aware of what difficulties HCP may have when recruiting patients to CHAMP?
   2. Are you aware if patients comply with submitting BP readings for 30 consecutive days?
4. Are you aware for how long do patients continue using CHAMP?

**FINAL THOUGHTS**

1. What do you think of the current implementation of CHAMP?
2. What would you do differently if you were to lead the CHAMP implementation process?

**Patients using CHAMP**

How are you? Have you eaten? Thank you for taking the time to talk to us today.

You are invited to this interview because you have high blood pressure, and you are enrolled (you could be enrolled in the future) onto CHAMP, which is a WhatsApp-based AI chatbot to support you in your journey of managing your blood pressure. Through this interview, we would like to understand how your experience with CHAMP has been (what would make you decide to use CHAMP if you were invited to participate in the program), and how to improve CHAMP further.

**ABOUT CHAMP**

1. Have you heard of CHAMP?

(IF YES) Could you please tell us what CHAMP is?

- 1. Who is chatting with you here?
  2. What do you think is the purpose of CHAMP?

1. (If Q1 YES) How did you hear about CHAMP?
   1. Did your care team offer you to enrol into CHAMP?
2. How long have you used CHAMP?
3. What would you say are the benefits of CHAMP to you?
4. Are you aware of other interventions to monitor your blood pressure?
   1. Have you used them?
   2. How does CHAMP compare with those interventions?
      1. Is CHAMP easier or more difficult to use than those interventions? In what ways (prompt them to elaborate)
      2. Are there any advantages or disadvantages of using CHAMP over the other interventions?
5. How do you find the experience of using CHAMP so far?
6. Could you please tell us more?
7. What do you like about CHAMP?
8. What do you not like about it?
9. If you could change something about CHAMP to make it work better for you, what would you change and why?
10. What other features will be useful?
11. Would you like to continue using CHAMP?
    1. Could you please tell us more? Why (not) would you like to continue using CHAMP?

**CHAMP ENROLLMENT AND USE**

1. How did you get enrolled into CHAMP?
2. Who told you about this programme?
3. Did you receive any training about how to use CHAMP?
4. (If NO) Would you have liked to receive some training on how to use CHAMP?

How would you like the training to be? What format? How long should the training last?

1. (If YES) Did you feel you needed it?

How was the training? Would you prefer the training to be done differently?

1. Could you show us how it works?
2. Do you experience any problems while using CHAMP?
3. Could you please tell us about these problems?
4. When you have problems using CHAMP, what do you do?
5. Are you aware that CHAMP has a help guide?
6. Do you know where to find the help guide in the chatbot?
7. What do you think of the information given in the help guide? Is it useful for you? Why/why not?
8. How often do you interact with CHAMP?
   1. Do you find the interaction engaging?
9. How could the interaction be improved?
10. When you signed up for CHAMP, you received reminders to measure your blood pressure every day and send the readings for 30 days. Did you manage to do it?
11. (If YES) Why do you think it was important to send the daily readings?
12. (If NO) Could you please tell us why it was difficult to send the daily BP readings?

**USING WHATSAPP**

1. What are your views about using WhatsApp for CHAMP?
2. If you were to design how CHAMP looks like, would you change anything?
   1. Could you please tell us more?

**MESSAGING (Bring a printed copy of current messages)**

1. What do you think about the messages CHAMP sends you?
2. Are the messages useful?
3. Are the messages clear and easy to understand?
4. Do you know how to respond to CHAMP questions?
5. If you were given the chance, would you like to make changes to the messages CHAMP sends you?

(If YES), what types of changes would you like to make?

**COST**

1. At the moment CHAMP is free of charge for patients. Would you use CHAMP if you had to pay for it?

(If YES) How much would you be willing to pay?

**ABOUT HYPERTENSION**

1. What do you know about hypertension?
2. What do you know about how to manage or treat hypertension?
3. What are your views about how well are you managing your hypertension?
4. Who else or what else can help you manage your hypertension?

**Patients who are not using CHAMP** Before we start with the questions:

1. Have you heard of CHAMP?

(if YES) Could you please tell us what CHAMP is?

1. Who is chatting with you here?
2. What do you think is the purpose of CHAMP?

(IF NO/patient explanation of CHAMP is not correct) CHAMP is a chatbot, a computer program delivered in WhatsApp that helps you monitor your blood pressure. CHAMP sends messages to remind you to measure your blood pressure, and help you decide if you have to see the doctor or not if your blood pressure is very high or very low.

1. (If Q1 YES) How did you hear about CHAMP?2
   1. Did your care team offer you to enrol into CHAMP?
   2. (If YES) Did you accept enrolling?
      1. (If YES) Why did you decide to stop using CHAMP?
      2. (If NO) Would you be interested in enrolling and using CHAMP?

**WILLIGNESS TO USE A CHATBOT FOR BLOOD PRESSURE MONITORING**

1. Would you be willing to use a chatbot/computer program to help you monitor your blood pressure?
   1. What would you say are the benefits of using a chatbot to monitor blood pressure?

(Prompts) reminder to monitor/ advantage over writing in a piece of paper/ better communication with care team about BP readings

1. What would you say are the disadvantages of using a chatbot to monitor blood pressure?

(Prompts) no trust in technology/ impersonal/ worries about privacy and data security

1. CHAMP is one of many digital tools that help people manage their blood pressure. Are you aware of other interventions here in Singapore/NUP to monitor your blood pressure?
2. Have you used them?
3. (If YES) What was your impression about using this tool?

**USING WHATSAPP**

1. What are your views about receiving WhatsApp messages from a chatbot to monitor your blood pressure?

**VIEWS ON CHAMP**

1. (To show participant CHAMP in a phone/printed prototypes) This is what CHAMP looks like. Could you please tell me what do you think about it?
2. What do you think about the messages CHAMP sends?
3. Are these messages clear and easy to understand?
4. Would you know how to respond to the questions?
   1. Would you know what systolic and diastolic BP mean?
5. How often would you like to receive messages from CHAMP/a WhatsApp chatbot?
   1. Would daily messages be adequate?
6. At the moment CHAMP is free of charge for patients. Would you use CHAMP if you had to pay for it?
7. (If YES) How much would you be willing to pay?

**ABOUT HYPERTENSION**

1. What do you know about hypertension?
2. What do you know about how to manage or treat hypertension?
3. What are your views about how well are you managing your hypertension?
4. Who else or what else can help you manage your hypertension?
