# Supplementary material for: Implementation of a Digital Health Intervention (CHAMP) for Self-Monitoring of Hypertension: Protocol for 3 Interlinked Implementation Studies
Source: JMIR Res Protoc. 2025 Oct 17;14:e72942. doi: 10.2196/72942 (PMC12579285; doi:10.2196/72942)
Supplement: Multimedia Appendix 3 [file resprot_v14i1e72942_app3.doc]

**Multimedia Appendix 3**

**Organizational Readiness for Implementing Change (ORIC)**

| 1 | 2 | 3 | 4 | 5 |
| --- | --- | --- | --- | --- |
| Disagree | Somewhat  Disagree | Neither Agree nor Disagree | Somewhat  Agree | Agree |

| 1. People who work here feel confident that the organization can get people invested in implementing this change. | 1 2 3 4 5 |
| --- | --- |
| 1. People who work here are committed to implementing this change. | 1 2 3 4 5 |
| 1. People who work here feel confident that they can keep track of progress in implementing this change. | 1 2 3 4 5 |
| 1. People who work here will do whatever it takes to implement this change. | 1 2 3 4 5 |
| 1. People who work here feel confident that the organization can support people as they adjust to this change. | 1 2 3 4 5 |
| 1. People who work here want to implement this change. | 1 2 3 4 5 |
| 1. People who work here feel confident that they can keep the momentum going in implementing this change. | 1 2 3 4 5 |
| 1. People who work here feel confident that they can handle the challenges that might arise in implementing this change. | 1 2 3 4 5 |
| 1. People who work here are determined to implement this change. | 1 2 3 4 5 |
| 1. People who work here feel confident that they can coordinate tasks so that implementation goes smoothly. | 1 2 3 4 5 |
| 1. People who work here are motivated to implement this change. | 1 2 3 4 5 |
| 1. People who work here feel confident that they can manage the politics of implementing this change. | 1 2 3 4 5 |

**Readiness Thinking Tool**

| Motivation | Degree to which an organization wants the innovation to happen. | Disagree | Partially Agree | Strongly Agree | Unsure |
| --- | --- | --- | --- | --- | --- |
| **Relative Advantage** | This innovation seems better than what we are currently doing. |  |  |  |  |
| **Compatibility** | This innovation fits with how we do things. |  |  |  |  |
| **Simplicity** | This innovation seems simple to use. |  |  |  |  |
| **Ability to Pilot** | This innovation can be tested and experimented with. |  |  |  |  |
| **Observability** | We have the ability to see that this innovation is leading to outcomes. |  |  |  |  |
| **Priority** | This innovation has a high level of importance compared to other things we do. |  |  |  |  |
| Innovation-specific Capacity | What is needed to make this particular innovation happen. |  |  |  |  |
| **Innovation-specific Knowledge & Skills** | We have sufficient abilities to do the innovation. |  |  |  |  |
| **Champion** | There is a well-connected person who supports and models this innovation. |  |  |  |  |
| **Supportive Climate** | We have the necessary supports, processes, and resources to enable this innovation. |  |  |  |  |
| **Inter-organizational Relationships** | We have the necessary relationships between organizations that support this innovation. |  |  |  |  |
| **Intra-organizational Relationships** | We have the necessary relationships within organization that support this innovation. |  |  |  |  |
| General Capacity | The organization’s overall functioning. |  |  |  |  |
| **Culture** | We have clear norms and values of how we do things here. |  |  |  |  |
| **Climate** | People have a strong sense/feeling of being part of this organization. |  |  |  |  |
| **Innovativeness** | Our organization is open to change in general. |  |  |  |  |
| **Resource Utilization** | Our organization has the ability to acquire and allocate resources including time, money, effort, and technology. |  |  |  |  |
| **Leadership** | Our organization has effective leaders. |  |  |  |  |
| **Internal Operations** | Our organization has effective communication and teamwork. |  |  |  |  |
| **Staff Capacities** | Our organization has enough of the right people to get things done. |  |  |  |  |
| **Process Capacities** | Our organization has the ability to plan, implement, and evaluate. |  |  |  |  |
